# Design of Precision-Aware Subthreshold-Based MOSFET Voltage Reference

**DOI:** 10.3390/s22239466

**Published:** 2022-12-03

**Authors:** Shuzheng Mu, Pak Kwong Chan

**Affiliations:** School of EEE, Nanyang Technological University, Singapore 639798, Singapore

**Keywords:** voltage reference, bandgap reference, temperature compensation, operational amplifier, PVT variation, process sensitivity, temperature coefficient, sensor circuit

## Abstract

A new precision-aware subthreshold-based MOSFET voltage reference is presented in this paper. The circuit was implemented TSMC−40 nm process technology. It consumed 9.6 μW at the supply voltage of 1.2 V. In this proposed work, by utilizing subthreshold-based MOSFET instead of bipolar junction transistor (BJT), relatively lower power consumption was obtained in the design while offering comparable precision to that offered by its BJT counterpart. Through the proposed second-order compensation, it achieved the temperature coefficient (T.C.) of 3.0 ppm/°C in the TT corner case and a 200-sample Monte-Carlo T.C. of 12.51 ppm/°C from −40 °C to 90 °C. This shows robust temperature insensitivity. The process sensitivity of $V_{ref}$ without and with trimming was 2.85% and 0.75%, respectively. The power supply rejection (PSR) was 71.65 dB at 100 Hz and 52.54 dB at 10 MHz. The Figure-of-Merit (FOM) for the total variation in output voltage was comparable with representative BJT circuits and better than subthreshold-based MOSFET circuits. Due to low T.C., low process sensitivity, and simplicity of the circuit architecture, the proposed work will be useful for sensor circuits with stringent requirements or other analog circuits that require high precision applications.

## 1. Introduction

**Figure 1:**
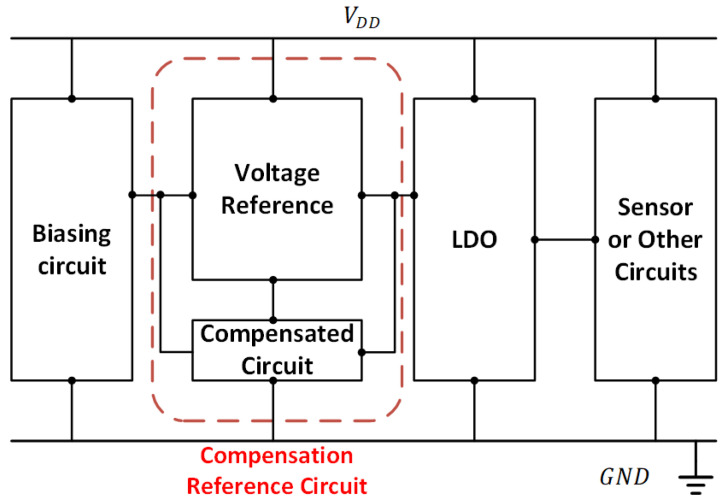
Exemplary Electronic System with High-order Temperature Compensated Voltage Reference.

With CMOS technology being scaled down to deep sub-micron levels, the design of a precise voltage reference with a small temperature coefficient (*T*.*C*.) under a wide temperature range has become a challenging topic. For analog circuits and digital circuits, especially in the sensor applications, the voltage reference will influence the performance and accuracy of the entire system directly. Therefore, a quality voltage reference with high-order temperature compensation will be needed by many high-performance electronic systems, such as the example as depicted in Figure 1. In general, the voltage reference design can be classified into bandgap reference (BGR) circuits [1,2,3,4,5,6,7,8,9,10,11,12] and subthreshold-based MOSFET voltage reference circuits [13,14,15,16]. They are utilized in applications that include analog-to-digital converters (ADC) [11], digital-to-analog converters (DAC) [12], sensor applications [13,14,15,16], and so forth.

The bipolar junction transistor (BJT), or parasitic BJT in CMOS technology, is the most common device in BGR circuit design, due to process characteristics [17] that are able to provide reasonably stable voltage. Unfortunately, the drawbacks of BJTs are obvious in many circumstances. First, BGR circuits often need higher supply voltages because of their turn-on voltage of about 0.7 V between base and emitter. Hence, being dependent on circuit architecture, the circuit’s minimum supply voltage may be pushed higher due to the high value of the turn-on voltage. Second, the collector current of a BJT is usually higher than the drain current of a MOSFET [18]. As such, BJT-based BGRs have to consume more power. This is mainly because the current gain β is a function of the collector current $I_{C}$. This suggests that BGRs with BJTs have to be designed with optimal collector currents to attain good thermal stability, as well as reduced circuit sensitivity. As a result, significant effort around the optimization design tradeoff is often encountered for thermal stability and power consumption. Third, for very large-scale integration (VLSI) circuit design today, parasitic vertical BJTs are popularly used, but they suffer from some performance pitfalls, such as poor current gain, low output resistance, big series base resistance, and so forth [19]. These unfavorable attributes may lead to complicated circuit realization [20,21] for improved compensation performance at the expense of increased power consumption. Regarding other design approaches to tackle the lower-power low-voltage design concern, some researchers proposed the picowatt 2-Transistor (2T) voltage reference [22]. This simple topology produces good reference voltage without using any amplifiers. Not only does it take up less chip area, it consumes ultra-low power consumption. Hence, it is very attractive for sensor applications which rely on limited energy source, such as biomedical applications or wireless sensor nodes. As reported by [22], the average T.C. after trimming was about 29 ppm/°C. This is considered acceptable in many applications. However, this type of reference circuits generally yields too low in the output voltage, which ranges between 100 and 200 mV. Despite the stacked design being able to scale the output voltage, both T.C. and output voltage variation will be increased as well. Alternatively, in order to generate the reference voltage with low T.C. and larger output voltage while addressing low power consumption at the same time, the replacement of BJTs with the subthreshold-based MOSFETs in the voltage reference circuits [23,24] are popular in design. Theoretically, the drain current for a sub-threshold MOSFET can be expressed as

(1) $$I_{D}=I_{D0}\exp\left(\frac{V_{GS}-V_{THN}}{nV_{T}}\right)$$

where $I_{D0}=\left(n-1\right)\mu_{n}C_{ox}\frac{W}{L}{\left(V_{T}\right)}^{2}$, n is the subthreshold slop factor, for which $n=\frac{C_{ox}+C_{J0}}{C_{ox}}\approx 1.5$ in CMOS technology, $\mu_{n}$ is the carrier mobility for electrons, $C_{ox}$ is the gate capacitor, and $V_{T}=\frac{KT}{q}$ is the thermal voltage, with the assumption that $V_{DS}>4V_{T}$. As can be seen, the MOSFET exhibits the BJT’s exponential behavior. The key advantages are low bias current and small $V_{GS}$ value, which lead to lower supply design and low power dissipation with respect to its BJT counterpart. By adding the second-order temperature compensation in the circuit [24], the T.C. can be pushed lower. However, it suffers from a higher variation in output reference voltage, due to relatively higher sensitivity to process variation using this circuit topology.

This raises the motivation of this work to propose the design and implementation of an improved voltage reference circuit that addresses the power consumption comparable to the subthreshold-based MOSFET designs, while the total precision is comparable with that of the BJT-based designs. In this work, a new subthreshold-based voltage reference that offers simpler circuit architecture with respect to that of the reported works of [2,3,4,5,6,7,18,19,20,21,22,23,24,25,26,27] is introduced. It permits good precision, which is achieved by low T.C., low process sensitivity, and simple trimming means while consuming low power.

Section 1 gives the introduction. Section 2 reviews the representative voltage reference circuits at different types. Section 3 presents the proposed voltage reference in conjunction with its design, analysis, and implementation. Section 4 presents the results and discussions of the voltage reference and its performance comparison with the reported prior works using parasitic BJT design or sub-threshold MOSFET design. This is then followed by a conclusion in Section 5.

## 2. Representative Voltage References

**Figure 2:**
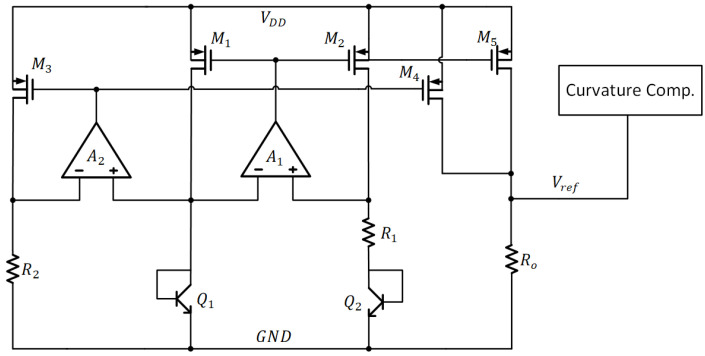
The Current-mode BG Circuit with Low T.C.

Figure 2 shows a current-mode BGR [21] employing the curvature compensation. The PTAT current is produced by $Q_{1}$, $Q_{2}$, $R_{1}$, and $A_{1}$, whereas the CTAT current is produced by $Q_{1}$, $A_{2}$, and $R_{2}$. Through the associated PMOS current mirrors, the CTAT current and PTAT current are combined, and thus, the reference voltage, $V_{ref}$, is generated through the resistor$\;R_{0}$. For improving the precision, the auto-zero technique is realized to cancel the offset of operational amplifiers $A_{1}$ and $A_{2}$. Besides, the multi-sectional curvature compensation is employed to correct the high-order temperature coefficient. This leads to an average T.C. of 8.75 ppm/°C and a process sensitivity of 0.54%. Although the output has good precision, the circuit consumes significant current consumptions of 120 μA, which are considerably high.

**Figure 3:**
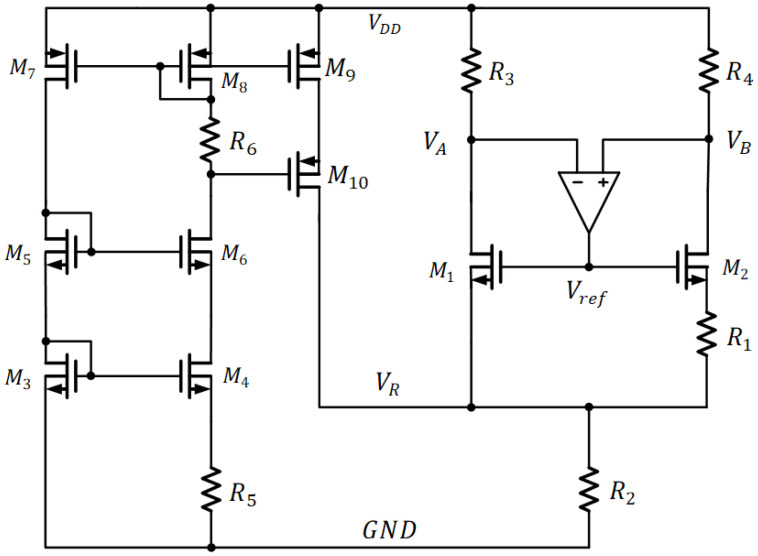
The Sub-1V MOS Threshold Voltage Circuit.

A sub-1V MOS threshold voltage circuit was proposed [24] to reduce the power consumption through the subthreshold-based design while addressing low supply voltage and low T.C. using second-order temperature compensation. Figure 3 shows the circuit implementation. $M_{1}$ and $M_{2}$ work in the sub-threshold region. $V_{A}$ is forced to be equal to $V_{B}$ though an amplifier in a loop. The MOSFET’s $V_{GS}\left(T\right)$ comprises the dominant first-order negative T.C. and small positive T.C. in second-order form. The PTAT voltage contributed by $\Delta V_{GS}\left(T\right)$, $R_{1}$, and $R_{2}$ will cancel the first-order negative T.C. arising from $V_{GS}\left(T\right)$. On the other hand, the positive T.C. of resistor $R_{5}$ in the PTAT current generator and the negative T.C. of resistor $R_{2}$ in the reference circuit will form the small negative quadratic T.C. term to counteract the positive quadratic T.C. term in the $V_{GS}\left(T\right)$. As a result, the average T.C. is 40 ppm/°C without trimming, and the output process sensitivity is 3.4 % while consuming 290 nW. Due to the mismatch effect of current mirrors in the PTAT current generator, the PTAT compensating current will be deviated. As such, it may be difficult to push the T.C. lower.

**Figure 4:**
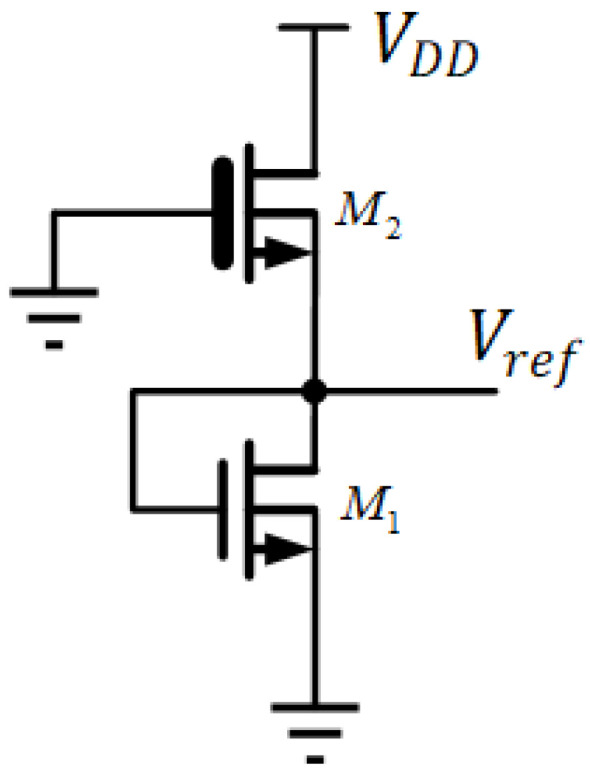
2-Transistor Voltage Reference.

2-Transistor voltage reference [22] in Figure 4 is another representative design in low power circuits. This topology consists of two transistors, $M_{1}$ and $M_{2}$, which operate in the sub-threshold region. $M_{1}$ is a native transistor, and it functions as the current generator with exponential VI conversion. $M_{2}$ serves as an active load with an inverse IV characteristic. As a result, a constant reference output voltage can be established through both nonlinear VI and IV conversion processes. Due to first-order temperature compensation, the average T.C. is about 62 ppm/°C without trimming. After trimming, the average T.C. is about 29 ppm/°C. This T.C. performance metric generally meets many applications. Although the circuit features an ultra-low power consumption of 2.2 pW at 0.5 V supply voltage, the generated output reference voltage is merely 176 mV, due to the diode transistor output. However, for designs with higher output voltage requirements, this may not be adequate. Nevertheless, the stacked topology can increase the output voltage in exchange for increased T.C. and higher variation in output voltage, due to increased circuit sensitivity.

## 3. Proposed Second-Order Temperature Compensated Voltage Reference

### 3.1. Temperature Compensation of Proposed Voltage Reference

**Figure 5:**
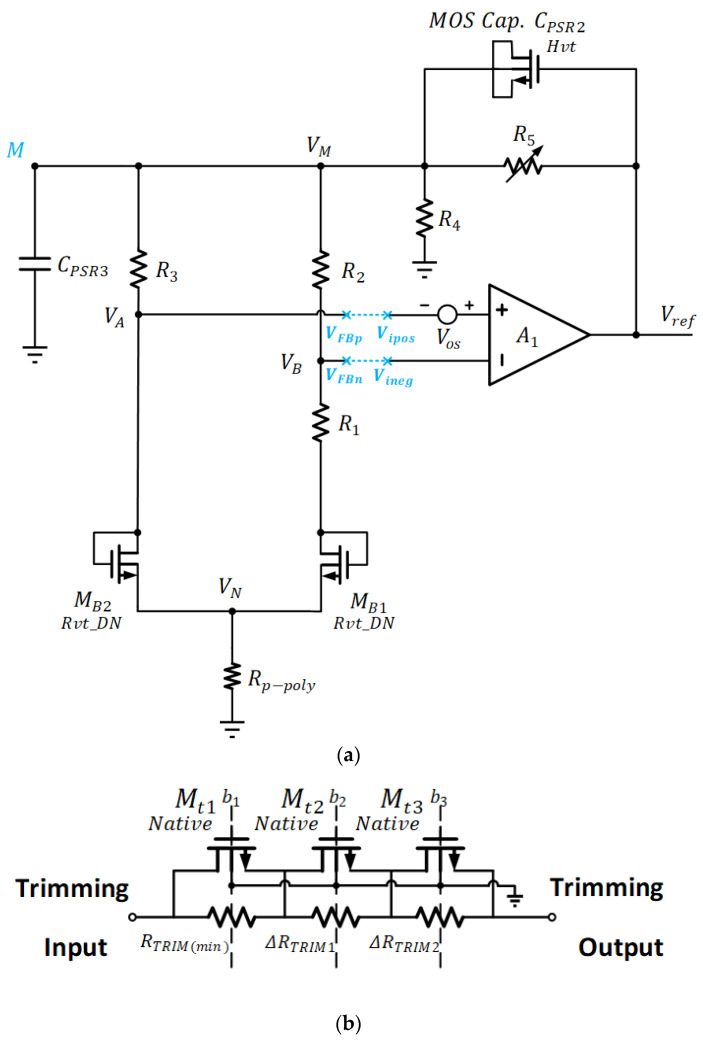
Proposed Voltage Reference Circuit with Second-Order Temperature Compensation: (**a**) Circuit Schematic (**b**) Binary-Weighted Trimming Circuit for $R_{5}$.

The proposed voltage reference circuit is depicted in Figure 5. In contrast to the topology [24] in Figure 3, the PTAT current source is embedded within the reference circuit, which leads to further simplification without a separate PTAT current generator and extra current mirror. The proposed voltage-mode voltage reference comprises an operational amplifier, two MOS transistors, four temperature compensation resistors having two types of T.C values, one resistor scaling network for output voltage, and two PSR capacitors. When referring to Figure 5, the reference circuit output can be obtained as:

(2) $$V_{ref}\left(T\right)=\left(\frac{\Delta V_{GSB}\left(T\right)R_{3}\left(T\right)}{R_{1}\left(T\right)}+\frac{\Delta V_{GSB}\left(T\right)R_{p-poly}\left(T\right)}{R_{1}\left(T\right)}+V_{GSB2}\left(T\right)\right)\left(1+\frac{R_{5}\left(T\right)}{R_{4}\left(T\right)}\right)$$

As indicated, $\frac{\Delta V_{GSB}\left(T\right)R_{3}\left(T\right)}{R_{1}\left(T\right)}$ and $\frac{\Delta V_{GSB}\left(T\right)R_{p-poly}\left(T\right)}{R_{1}\left(T\right)}$ are used to compensate the temperature effect of $V_{GSB2}\left(T\right)$. With reference to [24], for the MOSFET to operate at the sub-threshold region, $V_{GSB2}\left(T\right)$ exhibits a complementary-to-absolute-temperature (CTAT) characteristic. Hence, $V_{GSB2}\left(T\right)$ can be expressed as follows:

(3) $$V_{GSB2}\left(T\right)=V_{TH02}-\left(m-1\right)\frac{nk_{B}T_{r}}{2q}+\left[V_{GS2}\left(T_{r}\right)-V_{TH02}\right]\frac{T}{T_{r}}+\left(m-1\right)\frac{nk_{B}}{2qT_{r}}T^{2}$$

where $T_{r}$ is the reference temperature, $V_{TH02}$ is the intrinsic threshold voltage, m≈1.5−2 is the temperature exponent of mobility, and $k_{B}$ is the Boltzmann constant. From the above extrapolation, it can be interpreted that $V_{GSB2}\left(T\right)$ can be broken down into two temperature-dependent parts. $\left[V_{GSB2}\left(T_{r}\right)-V_{TH02}\right]\frac{T}{T_{r}}$ is the negative linear temperature component and $\left(m-1\right)\frac{nk_{B}}{2qT_{r}}T^{2}$ is the positive quadratic component. Similarly, $V_{GSB1}\left(T\right)$ can be expressed as follows:

(4) $$V_{GSB1}\left(T\right)=V_{TH01}-\left(m-1\right)\frac{nk_{B}T_{r}}{2q}+\left[V_{GS1}\left(T_{r}\right)-V_{TH01}\right]\frac{T}{T_{r}}+\left(m-1\right)\frac{nk_{B}}{2qT_{r}}T^{2}$$

For $M_{B1}$ and $M_{B2}$ operating in the sub-threshold region and having different aspect ratios, with$\;{\left(W/L\right)}_{1}$ $>{\left(W/L\right)}_{2}$, it can be proved that the difference of gate-to-source voltage [25] is given as:

(5) $$\Delta V_{GSB}=V_{GSB2}\left(T\right)-V_{GSB1}\left(T\right)=nV_{T}In\left(M\right)$$

where $M=\frac{{\left(W/L\right)}_{1}}{{\left(W/L\right)}_{2}}$, and n and $V_{T}=\frac{KT}{q}$. It can be observed that $\Delta V_{GSB}\left(T\right)$ cancels the first-order and second-order temperature-dependent terms in $V_{GSB1}\left(T\right)$ and $V_{GSB2}\left(T\right)$, which leaves a constant that has the PTAT property.

Consider $R_{1}\left(T\right)-R_{5}\left(T\right)$ as the same type of PTAT resistor (high resistive n-poly resistor) and $R_{p-poly}\left(T\right)$ as a CTAT resistor (high resistive p-poly resistor). The two types of temperature-dependent resistors are modelled as follows:

(6) $$R_{p-poly}\left(T\right)=R_{p-poly}\left(T_{r}\right)\left[1+\alpha \left(T-T_{r}\right)\right]$$

(7) $$R_{1}\left(T\right)=R_{1}\left(T_{r}\right)\left[1+\beta \left(T-T_{r}\right)\right]$$

where α is the T.C. of $R_{p-poly}$, β is the T.C. of $R_{1}$, and $T_{r}$ is room temperature, and it is a constant. From the Process Design Kit, α and β are obtained as $-4.7837\times 10^{-5}/K$ and $1.476\times 10^{-4}/K$, respectively. When accounting for the temperate effect for the ratio of resistors having different types, the temperature components can be partitioned into linear and negative quadratic terms. They are expressed as follows:

(8) $$f\left(T\right)=\frac{R_{p-poly}\left(T_{r}\right)}{R_{1}\left(T_{r}\right)}\left\{\left[2\left(\alpha -\beta \right)T_{r}+1\right]T+\left(\alpha -\beta \right)T^{2}+\left(\alpha -\beta \right)T_{r}{}^{2}\right\}$$

As calculated, $\left(\alpha -\beta \right)<0$. Therefore, by choosing the appropriate ratio in the $\frac{R_{p-poly}\left(T_{r}\right)}{R_{1}\left(T_{r}\right)}$ term, the generated negative quadratic temperature component is able to counteract the positive quadratic temperature component in$\;V_{GSB2}\left(T\right)$ as described in (4), based on Figure 5a. Of particular note, the negative linear T.C. term in$\;V_{GSB2}\left(T\right)$ will be compensated by the PTAT-related term as described in (5). The temperature compensation in dual terms permits low T.C. to be achieved.

$R_{4}\left(T_{r}\right)$ and $R_{5}\left(T_{r}\right)$ in Figure 5a form the voltage scaling network. By adjusting the value of resistor $R_{5}\left(T_{r}\right)$, the output voltage can be tuned from 0.65 V to 0.9 V. It is noted that the two capacitors $C_{PSR2}$ and $C_{PSR3}$ are used to build two RC filters. The goal is to improve the power supply rejection (PSR) of this voltage reference at high frequencies. When accounting for the first-order and second-order terms as discussed, $V_{ref}\left(T\right)$ is obtained as follows:

(9) $$V_{ref}\left(T\right)=\left\{\frac{nV_{T}In\left(M\right)R_{3}\left(T_{r}\right)}{R_{1}\left(T_{r}\right)}\;\;\;\;\;\;\;\;\;\;\;\;\;\;\;\;\;\;\;\;\;\;\;\;+\frac{nV_{T}In\left(M\right)R_{p-poly}\left(T_{r}\right)}{R_{1}\left(T_{r}\right)}\left\{\left[2\left(\alpha -\beta \right)T_{r}+1\right]T+\left(\alpha -\beta \right)T^{2}\;\;\;\;\;\;\;\;\;\;\;\;\;\;\;\;\;\;\;+\left(\alpha -\beta \right)T_{r}{}^{2}\right\}+V_{GSB2}\left(T\right)\right\}\left(1+\frac{R_{5}\left(T_{r}\right)}{R_{4}\left(T_{r}\right)}\right)$$

In this design, $M=\frac{{\left(W/L\right)}_{1}}{{\left(W/L\right)}_{2}}=8$, with channel length $L_{1}=L_{2}$, $n\approx 1.5,\;V_{T}=\frac{KT}{q}\approx 26\;mV$, $\frac{R_{3}\left(T_{r}\right)}{R_{1}\left(T_{r}\right)}\approx 2.15$, $\frac{R_{1}\left(T_{r}\right)}{R_{p-poly}\left(T_{r}\right)}=7.61$, and $V_{ref}\left(T\right)=800\;mV$. Table 1 lists the size of each component in the reference circuit. As observed, the advantage of this proposed design is that it requires only one trim for the$\;R_{5}$ value to get the precision output. For the SS corner, MOSFET transistors have thicker gate oxide layers and higher threshold voltage. According to (2), this leads to the increase in output voltage through the increase in $V_{GSB2}$. In order to reduce the unwanted increment due to process variation, the trimming resistor $R_{5}$ should be made small, with $R_{5SS}=R_{TRIM\left(min\right)}$ for this worse condition. Regarding the TT corner, $R_{5TT}=R_{TRIM\left(min\right)}+\Delta R_{TRIM1}$ will be used, due to the decrease in $V_{GSB2}$ with reference to the baseline resistance defined by the SS corner. Similarly, for the FF corner, MOSFET transistors have thinner gate oxide layers and lower threshold voltage, which causes the decrease in output voltage. At this juncture, $R_{5FF}=R_{TRIM\left(min\right)}+\Delta R_{TRIM1}+\Delta R_{TRIM2}$ is employed for compensating the drop in $V_{GSB2}$.

### 3.2. Analysis of Proposed Voltage Reference

#### 3.2.1. Core Circuit of Operational Amplifier

**Figure 6:**
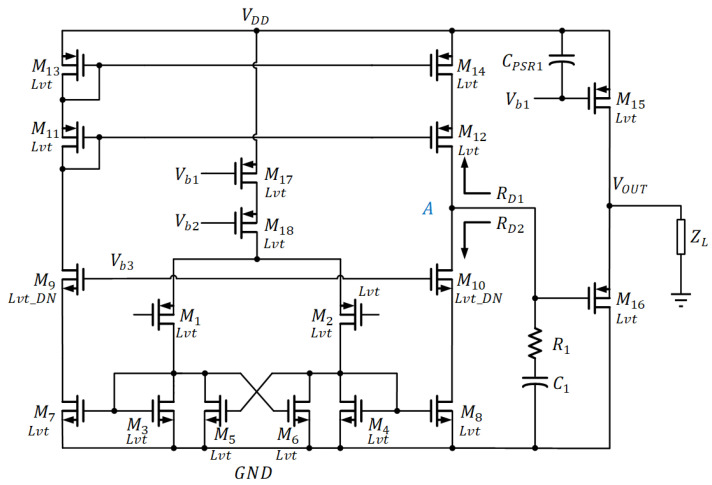
Operational Amplifier Circuit Topology.

The operational amplifier (op-amp), which is depicted in Figure 6, is a high-gain OTA topology with a source-follower output stage to drive a composite load $Z_{L}$ and a RC network for frequency compensation. The composite load $Z_{L}$ is basically the components, which comprise $M_{B1}$-$M_{B2}$, $R_{1}$-$R_{5}$, $R_{p-poly}$, $C_{PSR2}$, and $C_{PSR3}$, as shown in Figure 5a. All the transistors are biased in the sub-threshold region. Table 2 lists the size of each component in the operational amplifier. 

Due to relatively lower voltages developed by MOS devices than BJT counterparts, the pmos input stage is employed to handle low common-mode DC. With the differential pair $M_{1}$–$M_{2}$ driving the cross-coupled load $M_{3}$–$M_{6}$, it turns out that the effective impedance of the load is

(10) $$\frac{1}{\Delta g_{m}}=\frac{1}{\left(g_{m3}-g_{m5}\right)}=\frac{1}{\left(g_{m4}-g_{m6}\right)}$$

The introduction of positive feedback allows one of the impedances to turn negative. As such, higher gain can be obtained with the cross-coupled load through the difference between transconduce parameters. It is given that the positive feedback is less than negative feedback for ensuring stability. The rest of the high-gain arrangement comes from the cascode current mirror gain stage formed by $M_{7}$–$M_{14}$. This is then followed by source follower, comprising $M_{15}$–$M_{16}$, which permits the op-amp to have driving capability. High-gain is essential to obtain high PSR at low frequency. It also forces $V_{A}$ and $V_{B}$ very close to each other in Figure 5a. It is critical for precise voltage reference.

When referring to Figure 5a, the small-signal open-loop gain of the op-amp and the composite $Z_{L}$ are obtained as follows:

(11) $$A_{v0}=\frac{g_{m2}}{\Delta g_{m}}g_{m8}\frac{r_{o10}+R_{D1}}{1+g_{m10}r_{o10}}\frac{R_{D1}R_{D2}(g_{m10}r_{o10}+1)}{\left(R_{D1}+R_{D2}\right)r_{o10}}\frac{g_{m16}}{g_{ds15}+g_{ds16}+g_{m16}+\frac{1}{Z_{L}}}$$

(12) $$Z_{L}=\frac{sR_{5}R_{total}\left(C_{Mc1}+C_{P2}\right)+R_{5}+R_{total}}{s^{2}R_{5}R_{total}C_{Mc1}C_{P2}+s\left(R_{5}C_{Mc1}+R_{total}C_{P2}\right)+1}$$

where $R_{D1}$ is the equivalent resistance for $M_{12}$ and $M_{14}$, which equals to $g_{m12}r_{o12}r_{o14}+r_{o12}+r_{o14}$. $R_{D2}$ is the equivalent resistance for $M_{8}$ and $M_{10}$, which equals to $g_{m10}r_{o8}r_{o10}+r_{o8}+r_{o10}$. $Z_{L}$ is the composite load. $R_{total}$ is the equivalent resistor contributed by the components $R_{1}-R_{4},R_{p-poly},\;M_{B1},\;M_{B2}$.

Since $R_{D1},\;R_{D2}\gg \;r_{o10}$, and $g_{m16}\gg g_{ds15}+g_{ds16}+\frac{1}{Z_{L}}$ at low frequency, the open-loop gain of the op-amp can be simplified as

(13) $$A_{v0}=g_{m2}g_{m8}\frac{1}{\Delta g_{m}}\frac{R_{D1}}{r_{o10}}\frac{R_{D1}R_{D2}}{\left(R_{D1}+R_{D2}\right)}$$

#### 3.2.2. Basing Circuit of Operational Amplifier

**Figure 7:**
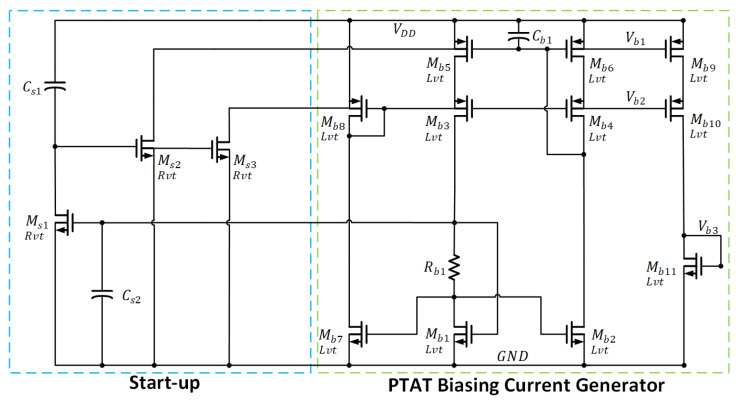
Biasing Circuit and Start-up Circuit.

Figure 7 shows a $g_{m}$-compensated biasing circuit with a capacitive start-up network. The circuit generates the PTAT current, which can maintain the gain and the gain bandwidth (GBW) of the sub-threshold-biased amplifier at high temperature. The PTAT current is given as

(14) $$I=\frac{\Delta V_{GS}\prime \left(T\right)}{R_{b1}\left(T\right)}$$

where $\Delta V_{GS}\prime \left(T\right)$ is the difference of $V_{GS}$ between $M_{b1}$ and $M_{b2}$. It is noted that when $R_{b1}\left(T_{r}\right)$ equals to the $\frac{1}{g_{m}}$, the basing circuit is independent of supply voltage. This enhances the PSR of the biasing circuit.

When $V_{DD}$ is powered on, the gate voltage of $M_{s2}$ and $M_{s3}$ are pulled high, due to the short circuit of $C_{s1}$ to the supply. As a result, the momentum current is drawn from the cascode current mirrors $M_{b5}$ and $M_{b6}$ through the start-up transistor $M_{s2}$. The cascode transistors, $M_{b3}$ and $M_{b4}$, are simultaneously turned on by the start-up transistor $M_{s3}$. This results in the bias currents being established in the core biasing circuit. Hence, $V_{GSb2}$ in $M_{b2}$ is then built up to establish the bias for the mirror transistor $M_{b7}$ and the cascode bias transistor $M_{b7}$. At the same time, $M_{s1}$ is turned on. When the capacitor $C_{s1}$ is charged through$\;M_{s1}$ and the supply voltage, the gate voltage for the start-up transistors $M_{s2}$ and $M_{s3}$ will go low so that both transistors go off when the biasing circuit reaches the steady state. The capacitor $C_{b1}$ is used to improve the PSR of the biasing circuit, and the capacitor $C_{s2}$ is used to add the latency for $M_{s1}$ to turn on when other parts of the circuit have completed the operation. Table 3 lists the size of each component in the biasing circuit. Table 3 lists the size of each component in the biasing circuit

#### 3.2.3. Frequency-Dependent Loop Gain Analysis of Voltage Reference

While designing the high-gain circuit, it may be more difficult to maintain the stability, due to the existence of a number of potential low-frequency poles. It is particularly significant when the biasing current of the circuit is made low in order to reduce power consumption. In this reference circuit design, the PSR capacitors are added to enhance high-frequency PSR at the expense of generating extra poles. To tackle the issue, a series of RC networks ($R_{1}$ and $C_{1}$) is employed at the output of cascode mirrors in the OTA such that an intentional zero is utilized to compensate for the unwanted phase shift produced by the PSR capacitor-related pole. As a result, the stability is not jeopardized.

When referring to voltage reference circuit in Figure 5a, consider $R_{5}$ and MOS capacitor $M_{c1}$ with a capacitance of $C_{PSR2}$, where the equivalent impedance is $Z_{1}=\frac{R_{5}}{sR_{5}C_{PSR2}+1}$. Besides, large resistors $R_{1}$, $R_{2}$, and $R_{p-poly}$ are employed while both $\frac{1}{g_{mB1}}$ and $\frac{1}{g_{mB2}}$ are large values for $M_{B1}$ and $M_{B2}$, respectively, under a small biasing current. As a result, $R_{4}$, by having a smaller resistance value, becomes the dominant resistor. As such, the equivalent impedance is approximated as $Z_{2}\approx \frac{R_{4}}{sR_{4}C_{PSR3}+1}$.

Thus, $V_{M}$ can be expressed by

(15) $$V_{M}\approx V_{OUT}\frac{Z_{2}}{Z_{1}+Z_{2}}=\left(V_{ineg}-V_{ipos}\right)A_{v}\frac{Z_{2}}{Z_{1}+Z_{2}}\;$$

(16) $$\frac{Z_{2}}{Z_{1}+Z_{2}}=\frac{sR_{4}R_{5}C_{PSR2}+R_{4}}{sR_{4}R_{5}\left(C_{PSR2}+C_{PSR3}\right)+R_{4}+R_{5}}$$

By breaking the negative feedback loop, the return signal is

(17) $$V_{FBn}\approx V_{M}\frac{\frac{1}{g_{mB1}}+\frac{R_{p-poly}}{2}+R_{1}}{\frac{1}{g_{mB1}}+\frac{R_{p-poly}}{2}+R_{1}+R_{2}}$$

Similarly, by breaking the positive feedback loop, the return signal is

(18) $$V_{FBp}\approx V_{M}\frac{\frac{1}{g_{mB2}}+\frac{R_{p-poly}}{2}}{\frac{1}{g_{mB2}}+\frac{R_{p-poly}}{2}+R_{3}}$$

By subtracting (18) from (17), the difference of the two outputs before the differential input of the op-amp becomes

(19) $$V_{FBn}-V_{FBp}\approx \left(V_{ineg}-V_{ipos}\right)A_{v}\frac{Z_{2}}{Z_{1}+Z_{2}}\left(\frac{\frac{1}{g_{mB1}}+\frac{R_{p-poly}}{2}+R_{1}}{\frac{1}{g_{mB1}}+\frac{R_{p-poly}}{2}+R_{1}+R_{2}}-\frac{\frac{1}{g_{mB2}}+\frac{R_{p-poly}}{2}}{\frac{1}{g_{mB2}}+\frac{R_{p-poly}}{2}+R_{3}}\right)$$

The negative feedback loop is made larger than the positive feedback loop in order to ensure the loop stability. Using the design parameter values, $Z_{L}$ is calculated to be about 107.9 kΩ in the unity-gain bandwidth, which is larger than the source follower’s output resistance, $\frac{1}{g_{m16}}$, which is of about 10.6 kΩ. Therefore, the influence of $Z_{L}$ in the high frequency is assumed to be negligible. The frequency-dependent open-loop gain of op-amp is given as:

(20) $$A_{v}=\frac{g_{m2}g_{m8}R_{D1}{}^{2}R_{D2}\left(\omega +\omega_{z1}\right)}{\Delta g_{m}r_{o10}\left(R_{D1}+R_{D2}\right)\left(\omega +\omega_{p1}\right)}$$

Consequently, the frequency-dependent loop gain of the reference circuit is obtained as follows:

(21) $$Loop\;gain=\frac{V_{FBn}-V_{FBp}}{V_{ineg}-V_{ipos}}\;\approx \frac{g_{m2}g_{m8}R_{D1}{}^{2}R_{D2}\left(\omega +\omega_{z1}\right)}{\Delta g_{m}r_{o10}\left(R_{D1}+R_{D2}\right)\left(\omega +\omega_{p1}\right)\left(\omega +\omega_{p2}\right)}\left(\frac{\frac{1}{g_{mB1}}+\frac{R_{p-poly}}{2}+R_{1}}{\frac{1}{g_{mB1}}+\frac{R_{p-poly}}{2}+R_{1}+R_{2}}-\frac{\frac{1}{g_{mB2}}+\frac{R_{p-poly}}{2}}{\frac{1}{g_{mB2}}+\frac{R_{p-poly}}{2}+R_{3}}\right)$$

There exist two significant low-frequency poles, which are located at node A and node M according to Figure 5a and Figure 6, respectively. The first dominant pole appears in node A. It is given as:

(22) $$\omega_{p1}=\frac{1}{(R_{out}+R_{1})C_{1}}\approx \frac{1}{R_{out}C_{1}}$$

where $R_{out}>R_{1}$ and $R_{out}\approx \left(g_{m10}r_{o8}r_{o10}\right)//\left(g_{m12}r_{o12}r_{o14}\right)$, which is the effective output resistance of the cascode mirrors in the OTA. The dominant zero at node A is given as:

(23) $$\omega_{z1}=\frac{1}{R_{1}C_{1}}$$

The second pole is located at node M in the voltage reference. It is given as:

(24) $$\omega_{p2}=\frac{R_{4}+R_{5}}{R_{4}R_{5}\left(C_{PSR2}+C_{PSR3}\right)}$$

**Figure 8:**
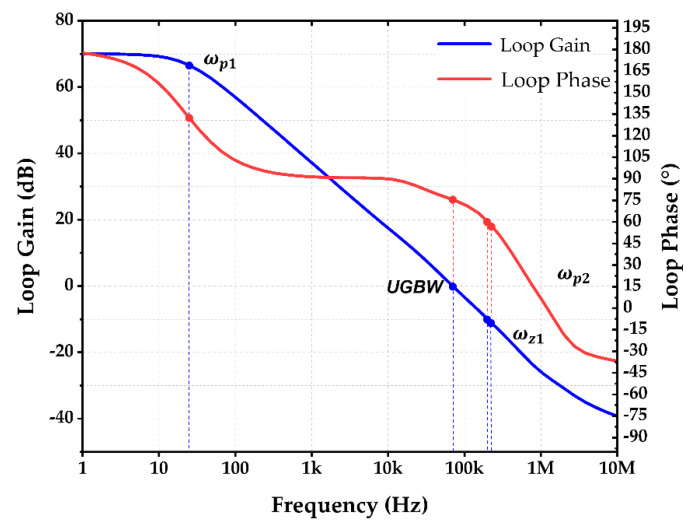
Loop Gain and Loop Phase of Reference Circuit.

Figure 8 depicts the plot of loop gain and phase against the frequency. The simulated phase margin (PM) and gain margin (GM) were obtained as 77° and −27 dB, respectively. The obtained two poles and one zero were then compared with the calculated results listed in Table 4. It was confirmed that the theoretical results matched the simulation results. The reference circuit was verified with good stability.

### 3.3. Offset Effect in Voltage Reference

Due to imperfect fabrication, non-ideal factors, such as offset, will influence the precision as well as the generation temperature-dependent effect in the reference circuit, which result in the degradation of the T.C. When referring to Figure 5a, in the presence of offset, $V_{os,in}$, at the input of high open-loop gain op-amp, we have:

(25) $$V_{B}=V_{A}+V_{os,in}$$

where $V_{A}=V_{GSB2}+V_{N}$ and $V_{B}=V_{GSB1}+V_{N}+I_{PTAT}\left(T\right)R_{1}$. Therefore, the output of the reference circuit is given as:

(26) $$V_{ref}\left(T\right)=\left(I_{PTAT}\left(T\right)R_{3}+V_{GSB2}\left(T\right)+2\times I_{PTAT}\left(T\right)R_{p-poly}\right)\left(1+\frac{R_{5}}{R_{4}}\right)$$

Since $I_{PTAT}\left(T\right)=\frac{\Delta V_{GS}\left(T\right)+V_{os,in}}{R_{1}}$, (26) can be re-written as follows:

(27) $$V_{ref}\left(T\right)=\left(\frac{\Delta V_{GS}\left(T\right)}{R_{1}}R_{3}+V_{GSB2}\left(T\right)+2\times \frac{\Delta V_{GS}\left(T\right)}{R_{1}}R_{p-poly}\right)\left(1+\frac{R_{5}}{R_{4}}\right)+\left(2\frac{V_{os,in}}{R_{1}}R_{p-poly}+\frac{V_{os,in}}{R_{1}}R_{3}\right)\left(1+\frac{R_{5}}{R_{4}}\right)$$

As can be seen, the non-ideal offset will be amplified, causing the degradation of accuracy and the T.C. of the reference circuit. To reduce the drawback, several issues can be addressed. To reduce the random offset, the aspect ratio W/L, area WL, and channel length L for the input transistor pair, current source transistors, and current mirror loads are made bigger whenever possible. This can reduce the mismatch in threshold voltage as well as the transconductance parameter, which are described by [25] as follows:

(28) $$\Delta V_{TH}=\frac{A_{VTH}}{\sqrt{WL}}$$

(29) $$\Delta \left(\mu C_{ox}\frac{W}{L}\right)=\frac{A_{K}}{\sqrt{WL}}$$

where $A_{VTH}$ and $A_{K}$ are process constants derived from the experiment. Their sizes are detailed in Table 2. In addition, the systematic offset was also addressed. This was done by balancing the nodal potential at A to the nodal potential of $M_{11}$ in op-amp, as depicted in Figure 6.

**Figure 9:**
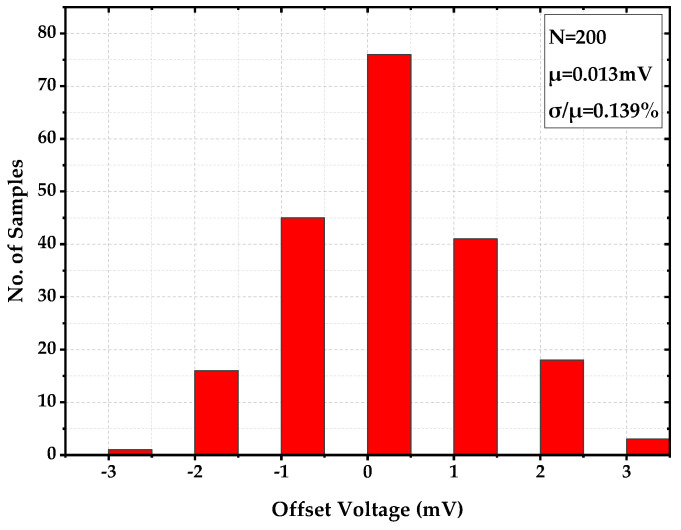
Offset Voltage of Op-Amp with 200-Sample Monte-Carlo Simulation.

For the offset evaluation, the op-amp was conducted with 200-sample Monte-Carlo simulation. Figure 9 shows the histogram of the offset, with a mean value of 0.013 mV and a process sensitivity of 0.139%. This verified that the offset had no significant impact on the reference circuit at the expense of increasing the size of critical devices. This is a particularly important design approach and design tradeoff for the precision voltage reference.

## 4. Results and Discussions

**Figure 10:**
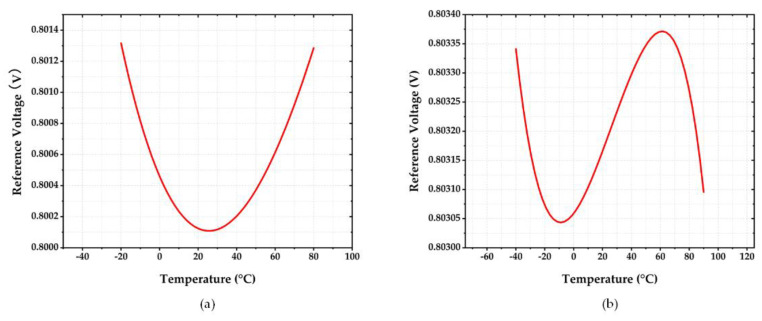
T.C. of $V_{ref}$ in the TT Corner Case (**a**) Circuit with 1st-order temperature compensation (**b**) Circuit with 2nd-order temperature compensation.

Having been implemented in TSMC 40 nm CMOS technology, the proposed reference circuit operated at a supply voltage of 1.2 V and a total current consumption of 8 μA. This yielded the total power consumption of 9.6 μW that comprised the power consumption contributed by the voltage reference circuit, the operational amplifier, and the bias circuit. For first-order temperature compensation, the obtained T.C. was 15.09 ppm/°C as shown in Figure 10a. For the second-order temperature compensation, the obtained T.C. became 3.002 ppm/°C for the temperature range from −40 °C to 90 °C as shown in Figure 10b. Both cases gave the reference output voltages of about 800 mV.

**Figure 11:**
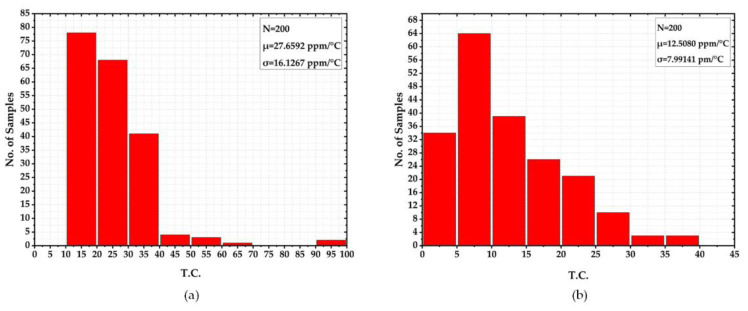
Monte-Carlo Simulation of T.C. (**a**) Circuit with 1st-order temperature compensation (**b**) Circuit with 2nd-order temperature compensation.

In order to evaluate T.C. sensitivity with respect to the variation in circuit and process parameters, two 200-sample Monte-Carlo simulations were conducted for the circuit with the first and second-order temperature compensations. Figure 11a,b illustrate the histograms of both results. As can be seen, the second-order compensated circuit in Figure 11b displayed the relatively lower T.C. values, with a mean value of 12.5 ppm/°C and standard deviation of 7.99 ppm/°C as compared to that of 27.65 ppm/°C and 16.13 ppm/°C, respectively, for the first-order temperature compensation. The results confirmed the effectiveness of the proposed temperature compensation method for the reference circuit.

**Figure 12:**
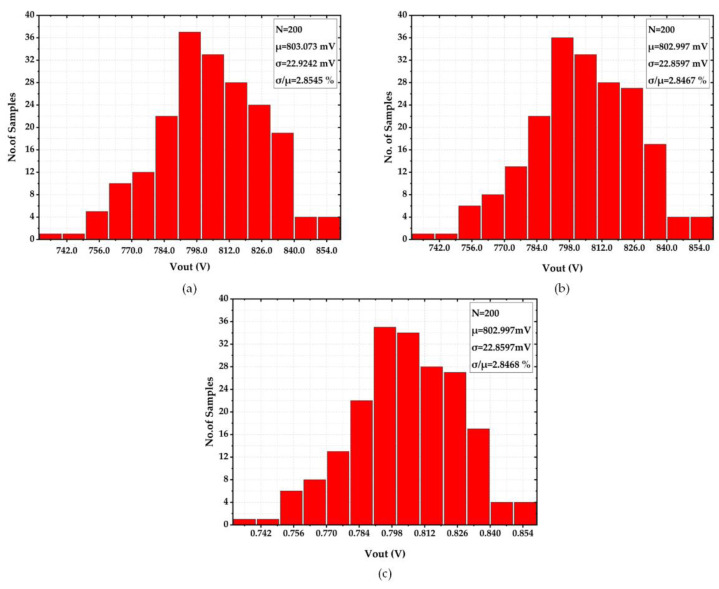
Monte-Carlo Simulation of Process Sensitivity (**a**) at −40 °C (**b**) at 27 °C (**c**) at 90 °C..

Besides, the precision of the voltage reference was also evaluated with a 200-sample Monte-Carlo simulation on the process sensitivity of the $V_{ref}$ at temperatures of −40 °C, 27 °C, and 90 °C. The respective histograms are depicted in Figure 12a–c. As can be seen, $V_{ref}$ varied very little in value and was about 803 mV, and the process sensitivity $\frac{\sigma }{\mu }$ was 2.85 % in the three temperature cases. This verified that the output voltage had good precision, low variation in process sensitivity, and high thermal stability.

**Figure 13:**
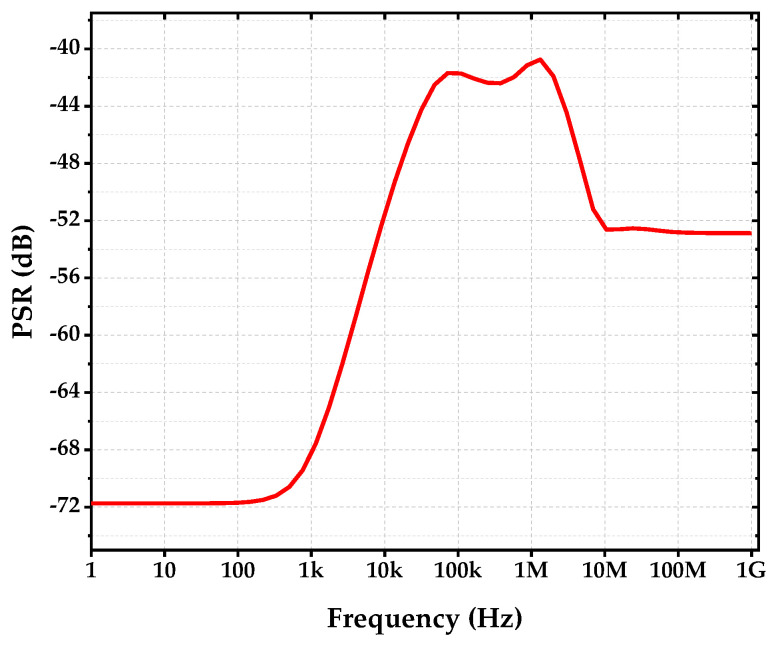
PSR of Proposed Reference Circuit.

Figure 13 shows the PSR of the proposed voltage reference circuit. As can be observed, the low-frequency (100 Hz) PSR gave −71.69 dB, which was due to the design of high open-loop gain op-amp, together with the use of source-follower to isolate the loading effect of resistors by the reference circuit. Besides, the gm-compensated biasing technique and high-frequency suppressing capacitor $C_{b1}$ were employed in the biasing circuit of the op-amp. This offered good rejection to supply noise. On top of that, the capacitor, $C_{PSR1}$, was added in the op-amp for high-frequency suppressing. At the juncture, two capacitors, $C_{PSR2}$ and $C_{PSR3}$, were added to form RC filters to reduce the supply noise of the reference circuit. This yielded at least a minimum of −40 dB on the PSR across the entire high-frequency range of the circuit. This is sufficient for many circuit applications.

**Figure 14:**
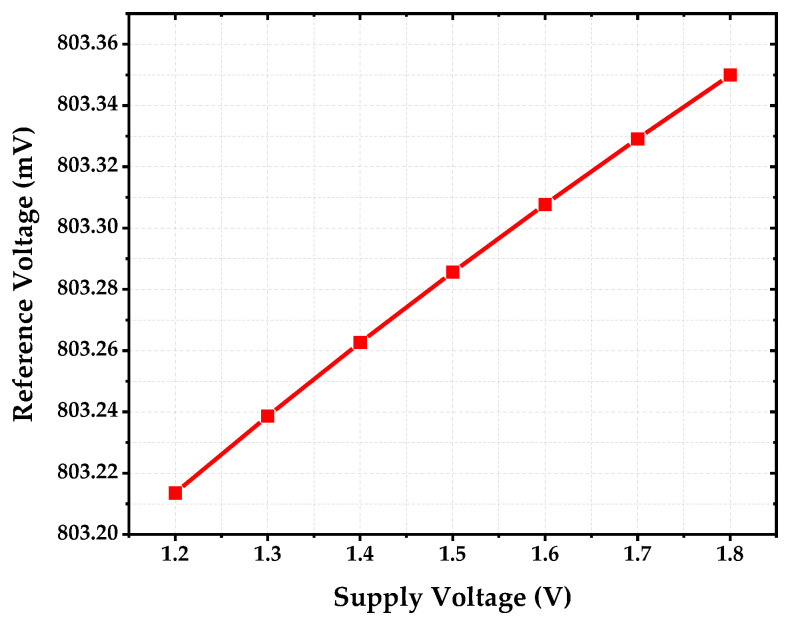
Line Sensitivity of Proposed Reference Circuit from 1.2 V to 1.8 V.

Figure 14 shows the line sensitivity plot of the proposed reference circuit with the supply voltage ranging from 1.2 V to 1.8 V. The obtained line sensitivity was 0.028 %/V, which is regarded as an acceptable value. This correlated with the performance of the low-frequency PSR.

**Figure 15:**
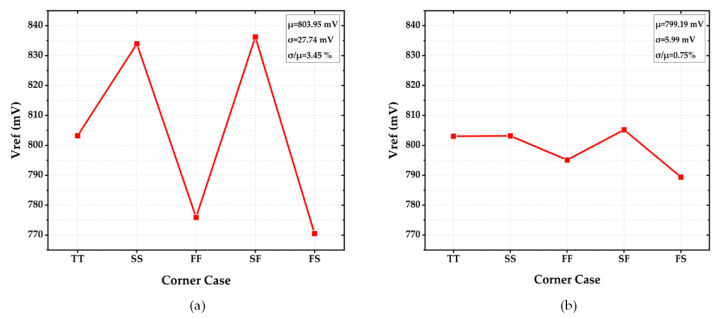
$V_{ref}$ at Different Corners, 27  °C (**a**) Before Trimming (**b**) After Trimming.

Figure 15a illustrates the untrimmed reference output voltage at corners TT, SS, FF, SF, and FS under the temperature of 27 °C. Since the targeted output voltage was 800 mV in design, it can be observed that data points up to a few tens of mV deviated from the reference value according to the type of process corner. Through 3-bit trimming of the $R_{5}$ in Figure 5a, the trimmed result was obtained in Figure 15b. As interpreted, the derivation with respect to the reference value of 800 mV was significantly reduced, leading to a maximum of 10.8 mV in deviation. This translated to an accuracy of 0.75%.

- Table 5 and Table 6 compare the performance of the proposed work with other representative reported works that include both types of parasitic BJT and sub-threshold MOSFET voltage references. Besides, a Figure-of-Merit (FOM) [26] performance metric was also utilized for evaluating the total PVT sensitivity of each voltage reference output. It is defined as follows:

(30) $$FOM=\left[\begin{array}{c} \left(Process\;Sensitivity\;\;\sigma /\mu \;\right)\\ +\left|\left(Line\;Sens.\right)\times 10\%V_{DD\left(min\right)}\right|\\ +\left(T.C.\;of\;V_{ref}\;\right)\times 100{}^{\circ}C \end{array}\right]$$

- The process sensitivity σ/μ pertains to the % output change due to process variation, the line sensitivity pertains to the % output change due to supply variation, and the T.C. of $V_{ref}$ pertains to the % output change due to temperature variation. The lower value of FOM indicates low sensitivity of the reference circuit output.
- When referring to the performance comparison of the BJT-based voltage references in Table 5, it can be seen that the proposed work offers slightly higher process sensitivity with respect to [6,21] but lower than [20] under no trimming condition. When trimmed, the proposed work offers comparable accuracy. Except for [6], it consumes lower power when compared with the majority of designs. However, the T.C. sensitivity obtained from the Monte-Carlo simulation is smaller than that of [6,20]. Regarding line sensitivity and low-frequency PSR, the obtained values are reasonably good. It is worth noting that the high-frequency PSR is very good in this work.

When referring to the performance comparison of subthreshold-based MOSFET voltage references in Table 6, the proposed work has the lowest T.C., and the average T.C. sensitivity obtained from Monte-Carlo simulation verified that the circuit is almost temperature insensitive. On top of that, without trimming, the process sensitivity of $V_{ref}$ is better than that of the reported works. With trimming, the process sensitivity offered an accuracy of up to 0.75%. Regarding the FOM without trimming, the proposed work offers better accuracy than the prior works. With trimming, the FOM of the proposed work reached 0.78%. This implied that the T.C. and line sensitivity of the $V_{ref}$ had no significant impact when compared to process sensitivity. However, the drawback was that the proposed circuit consumed relatively higher power than others. Hence, it was a tradeoff design between the BJT-based voltage reference and the subthreshold-based MOSFET voltage reference in terms of the tradeoff performance metrics relating to accuracy and power consumption. Finally, except for the 2T-based voltage reference, the proposed circuit has lower circuit complexity with respect to the representative subthreshold-based MOSFET voltage references. This is the reason why good thermal stability as well as accuracy were obtained when the second-order temperature compensation was proposed in this work.

## 5. Conclusions

A precision-aware subthreshold-based MOSFET voltage reference dedicated to the sensor and other analog circuit applications was presented in this paper. It was implemented in a TSMC 40 nm CMOS process, and the reference circuit operated at a 1.2 V supply. The paper showed that the proposed circuit could output a precision voltage though the simplicity of circuit architecture, the second-order temperature compensation, and the simple output voltage trimming feature. The circuit exhibited low T.C., low process sensitivity, and a good PSR, as well as reasonable power consumption and line sensitivity. When compared with the BJT reference circuits, the proposed work displayed lower power consumption and simpler quadratic temperature compensation to achieve low T.C., while the output accuracy was comparable when trimmed. When compared with the subthreshold-based MOSFET voltage references, the proposed work displayed lower T.C. and lower process sensitivity, with and without trimming.

## Figures and Tables

**Table 1:** Sizes of the Components in Voltage Reference Circuit.

Component	Size	Component	Size
$M_{B1}$	80/8 (μm/μm)	$R_{5}$	120 kΩ
$M_{B2}$	10/8 (μm/μm)	$R_{p-ploy}$	39 kΩ
$M_{t1}$	100/0.2 (μm/μm)	$C_{PSR2}$	20 pF
$M_{t2}$	100/0.2 (μm/μm)	$C_{PSR3}$	20 pF
$M_{t3}$	100/0.2 (μm/μm)	$R_{TRIM\left(min\right)}$	108 kΩ
$R_{1}$	300 kΩ	$\Delta R_{TRIM1}$	12 kΩ
$R_{2},\;R_{3}$	644 kΩ	$\Delta R_{TRIM2}$	8 kΩ
$R_{4}$	260 kΩ		

**Table 2:** Sizes of the Components in Operational Amplifier.

Component	Size	Component	Size
$M_{1},\;M_{2}$	60/3 (μm/μm)	$M_{15}$	40/6.7 (μm/μm)
$M_{3},\;M_{4}$	8/2 (μm/μm)	$M_{16}$	100/2 (μm/μm)
$M_{5},\;M_{6}$	7/2 (μm/μm)	$M_{17}$	1/3.5 (μm/μm)
$M_{7},\;M_{8}$	20/2 (μm/μm)	$M_{18}$	2.4/0.5 (μm/μm)
$M_{9},\;M_{10}$	50/1 (μm/μm)	$R_{1}$	210kΩ
$M_{11},\;M_{12}$	4/1 (μm/μm)	$C_{1}$	15pF
$M_{13},\;M_{14}$	10/2 (μm/μm)	$C_{PSR1}\;$	5pF

**Table 3:** Sizes of the Components in Biasing Circuit.

Component	Size	Component	Size
$M_{b1}$	12.8/2 (μm/μm)	$M_{b10}$	2.4/0.5 (μm/μm)
$M_{b2}$	10/2 (μm/μm)	$M_{s1}$	1/1 (μm/μm)
$M_{b3}$	4.8/0.5 (μm/μm)	$M_{s2}$	5/1 (μm/μm)
$M_{b4}$	1.2/0.5 (μm/μm)	$M_{s3}$	5/1 (μm/μm)
$M_{b5}$	2/3.5 (μm/μm)	$R_{b1}$	68.3kΩ
$M_{b6}$	0.5/3.5 (μm/μm)	$C_{s1}$	1pF
$M_{b7}$	15/2 (μm/μm)	$C_{s2}$	1.2pF
$M_{b8}$	2/20 (μm/μm)	$C_{b1}$	6pF
$M_{b9}$	1/3.5 (μm/μm)		

**Table 4:** Simulated and Calculated Values of Poles, Zeros and Unity-gain Bandwidth.

	$f_{p1}$	UGBW	$f_{p2}$	$f_{z1}$
Calculation (Hz)	23.08	70.53 k	304.87 k	317.46 k
Simulation (Hz)	22.30	85.40 k	316 k	331 k

**Table 5:** The Performance Benchmarks Against Voltage Reference with Parasitic BJT.

	[1]	[6]	[20]	[21]	This Work
**Year**	2015	2015	2018	2021	**2022**
**Technology (nm)**	130	90	500	130	**40**
**Temp. Range (°C)**	−40–120	0–70	−5–125	−40–150	**−40–90**
$V_{DD}$ **(V)**	1.2	1.15	2.1	3.3	**1.2**
$V_{OUT}$ **(V)**	0.735	0.72	1.196	1.160	**0.80**
**MC**$V_{OUT}$ **(V)**	NA	0.73	1.194	1.169	**0.80**
**Power (** μW **)**	120	0.58	38	120	**9.6**
**TT corner T.C. (ppm/** **°C** **)**	4.2	5.5	4.81	5.78	**3.00**
**MC T.C. (ppm/** **°C** **)**	NA	25	13.19	NA	**12.51**
**Line Sens. (%/V)**	NA	0.3	0.018	0.03	**0.028**
**PSR (dB) (100 Hz)**	−30	−51	−84	−82	**−71.69**
**PSR (dB) (10 MHz)**	NA	NA	NA	−20	**−52.54**
**Trimming Bits**	NA	NA	7	NA	**3**
**Process Sens.** **(**σ/μ**)w/o Trimming (%)**	NA	0.86	3.66	0.54	**2.85**
**Process Sens.** **(**σ/μ**)with Trimming (%)**	NA	NA	0.62	NA	**0.75**
**FOM w/o Trimming (%)**	NA	0.95	3.71	0.61	**2.88**
**FOM with Trimming (%)**	NA	NA	0.67	NA	**0.78**

**Table 6:** The Performance Benchmarks Against Subthreshold MOSFET-Only Voltage Reference.

	[27]	[24]	[18]	[19]	This Work
**Year**	2011	2014	2016	2021	**2022**
**Technology (nm)**	130	65	65	180	**40**
**Temp. Range (** **°** **C)**	−50–130	−40–90	−30–80	−40–130	**−40–90**
$V_{DD}$ **(V)**	0.7	0.75	1.1	0.9	**1.2**
$V_{OUT}$ **(V)**	0.501	0.477	0.47	0.261	**0.80**
**MC** $V_{OUT}$ **(V)**	NA	0.474	0.47	0.261	**0.80**
**Power (** μW **)**	0.21	0.29	2.64	1.8(nW)	**9.6**
**TT corner T.C. (ppm/** **°** **C)**	23.8	24	18.8	62	**3**
**Monte-Carlo T.C. (ppm/** **°** **C)**	NA	NA	21.7	NA	**12.51**
**Line Sens. (%/V)**	0.034	0.242	0.0071	0.013	**0.028**
**PSR (dB) (100 Hz)**	NA	−40	−54	−73.50	**−71.69**
**PSR (dB) (10 MHz)**	NA	−31	−43.5	NA	**−52.54**
**Trimming Bits**	NA	NA	NA	NA	**3**
**Process Sens. (** σ/μ **) w/o Trimming (%)**	NA	3.30	3.21	6.74	**2.85**
**Process Sens. (** σ/μ **) with Trimming (%)**	NA	NA	NA	NA	**0.75**
**FOM w/o Trimming (%)**	NA	3.56	3.40	7.36	**2.88**
**FOM with Trimming (%)**	NA	NA	NA	NA	**0.78**

## Data Availability

The study did not report any data.
